# Language barrier and its relationship to diabetes and diabetic retinopathy

**DOI:** 10.1186/1471-2458-12-781

**Published:** 2012-09-13

**Authors:** Yingfeng Zheng, Ecosse L Lamoureux, Pei-Chia Peggy Chiang, Ainur Rahman Anuar, Jie Ding, Jie Jin Wang, Paul Mitchell, E-Shyong Tai, Tien Y Wong

**Affiliations:** 1 Singapore Eye Research Institute, Singapore National Eye Centre, 11 Third Hospital Ave, #05-00, Singapore, 168751, Singapore; 2 State Key Laboratory of Ophthalmology, Zhongshan Ophthalmic Center, Sun Yat-sen University, #54 Xianlie Road, Guangzhou, 510060, China; 3 Centre for Eye Research Australia, University of Melbourne, the Royal Victorian Eye and Ear Hospital, 32 Gisborne Street, East Melbourne, VIC, 3002, Australia; 4 University of Malaya Eye Research Centre, Faculty of Medicine, University of Malaya, Kuala Lumpur, Malaysia; 5 Department of Ophthalmology, Faculty of Medicine, University of Malaya, Kuala Lumpur, 50603, Malaysia; 6 Centre for Vision Research, Department of Ophthalmology and Westmead Millennium Institute, University of Sydney, Sydney, NSW, 2145, Australia; 7 Department of Ophthalmology, Yong Loo Lin School of Medicine, National University of Singapore, 5 Lower Kent Ridge Road, Singapore, 119074, Singapore; 8 Department of Medicine, Yong Loo Lin School of Medicine, National University of Singapore, 5 Lower Kent Ridge Road, Singapore, 119074, Singapore

**Keywords:** English proficiency, Asian indians, Diabetes, Diabetic retinopathy, Visual impairment

## Abstract

**Background:**

Language barrier is an important determinant of health care access and health. We examined the associations of English proficiency with type-2 diabetes (T2DM) and diabetic retinopathy (DR) in Asian Indians living in Singapore, an urban city where English is the predominant language of communication.

**Methods:**

This was a population-based, cross-sectional study. T2DM was defined as HbA1c ≥6.5%, use of diabetic medication or a physician diagnosis of diabetes. Retinal photographs were graded for the severity of DR including vision-threatening DR (VTDR). Presenting visual impairment (VI) was defined as LogMAR visual acuity > 0.30 in the better-seeing eye. English proficiency at the time of interview was assessed.

**Results:**

The analyses included 2,289 (72.1%) English-speaking and 885 (27.9%) Tamil- speaking Indians. Tamil-speaking Indians had significantly higher prevalence of T2DM (46.2 vs. 34.7%, p < 0.001) and, among those with diabetes, higher prevalence of DR (36.0 vs. 30.6%, p < 0.001), VTDR (11.0 vs. 6.5%, p < 0.001), and VI (32.4 vs. 14.6%) than English speaking Indians. Oaxaca decomposition analyses showed that the language-related discrepancies (defined as the difference in prevalence between persons speaking different languages) in T2DM, DR, and VTDR could not be fully explained by socioeconomic measures.

**Conclusions:**

In an English dominant society, Tamil-speaking Indians are more likely to have T2DM and diabetic retinopathy. Social policies and health interventions that address language-related health disparities may help reduce the public health impact of T2DM in societies with heterogeneous populations.

## Background

Type 2 diabetes (T2DM) is one of the leading causes of mortality and disability worldwide [1]. People with T2DM have a substantial risk of diabetic retinopathy (DR), which, if left untreated, can lead to vision-threatening diabetic retinopathy (VTDR) and ultimately to visual impairment (VI) [2]. Recent evidence suggests that T2DM and its complications are not only determined by biological and lifestyle risk factors (e.g., obesity, hypertension, physical inactivity and unbalanced diet) [1], they are also affected by a broad range of social determinants (e.g., socioeconomic status and social support) [3].

Language barrier, among other social determinants, is known as an important factor predicting poorer health and barrier to care [4-10]. However, the impact of language barrier on diabetes and its ocular consequences have not previously been documented. Language barrier can be easily measured by a participant’s English proficiency during survey interview [11,12]. English proficiency during the interview is a functional measure determined by interviewers and therefore it is not subject to self-assessment bias [10,11].

Asian Indians are among the fastest growing ethnic groups in the United States, the United Kingdom, and in many Asian countries including Singapore. In Singapore, ethnic Indians (9.2%) is the nation’s third largest ethnic group, behind ethnic Chinese (74.1%) and Malays (13.4%) [13,14]. There are four major spoken languages (English, Mandarin, Malay and Tamil) in Singapore, with English being the official language for business, education and politics. However, more than 36% of Singaporean Indians reported Tamil as the language spoken most often at home [15]. The impacts of language skill on disease status has never been evaluated in Singapore, where the prevalence of diabetes was reported to be as high as 21.8% among those aged 50–59 and 32.4% among those aged 60 and over, and it disproportionately affects ethnic Indians more than any other [13,14]. Given the high prevalence of diabetes among Asian Indians and the unique multilingual nature of the country, we aimed to examine language-related disparities (defined as the difference in prevalence between persons normally speaking English and those normally speaking Tamil) in the prevalence of T2DM, DR and VI. Furthermore, if the effect of language is substantial, understanding why disparities exist between English and non-English speakers and the extent to which the language-related variation in health is due to variation in individual-level variables (e.g., biological risk factors, education, and income) may provide insights into public health strategies to reduce the burden and impact of T2DM. To answer this question, we used an Oaxaca decomposition method to decompose language-related disparities in the prevalence of T2DM, DR, VTDR and VI, and to quantify the contribution of individual-level variables.

## Methods

### Study population

The Singapore Indian Eye Study is a population-based cross-sectional study of Singaporean Indians aged 40 and over. Details of its methodology have been reported previously [16,17]. The Ministry of Home Affairs provided initial computer-generated lists of persons of Indian ethnicity residing in south-west Singapore. Of the 4,497 eligible subjects from the sampling frame, 3,400 (75.6%) participated. The study adhered to the Declaration of Helsinki and ethics approvals were obtained from the Singapore Eye Research Institute Institutional Review Board.

### Diabetes and diabetic retinopathy assessment

Based on American Diabetes Association’s diagnostic criteria, diabetes was defined as a self-reported previous diagnosis of the disease, or a hemoglobin A1c (Hba1c) ≥ 6.5%. A participant was considered to have type-1 diabetes if younger than 30 years when diagnosed with diabetes and received insulin therapy from diagnosis; other participants were considered to have T2DM.

Retinal photography was performed using a standardized protocol. After pupil dilation, one retinal photograph centered on the optic disc and another one on the macula were taken from both eyes using a digital retinal camera (Canon CR-DGi with a 10-D SLR back; Canon, Tokyo, Japan). Photographs were then sent to the University of Sydney, and retinopathy lesions were graded according to a scale modified from the Airlie House classification system [18,19]. Retinopathy severity was categorized into minimal non-proliferative diabetic retinopathy (NPDR; levels 15 and 20), mild NPDR (level 35), moderate NPDR (levels 43 and 47), severe NPDR (level 53), and proliferative diabetic retinopathy (PDR, levels more than 60). Diabetic macular edema was defined by a finding of hard exudates in the presence of MA and blot hemorrhage with one disc diameter from the foveal center or the presence of focal photocoagulation scars at the macular area. Those with diabetic macular edema were further divided into cases with clinically significant macular edema (CSME) and without CSME. CSME was defined by macular edema within 550 μm of the foveal center or if focal photocoagulation scars were present in the macular area. VTDR was defined as the presence of severe NPDR, PDR, or CSME. The severity scores of the worse of the two eyes were used for the individual. If the images in one eye were ungradable, the scores for the fellow eye were used to define these outcomes.

Visual acuity was measured using a logarithm of the minimum angle of resolution (logMAR) number chart (Lighthouse International, New York, NY). Presenting VI was defined as VA worse than 20/40 (logMAR > 0.30) in the better-seeing eye. Body Mass Index (BMI) was defined as weight divided by the square of height in meters (kg/m2). Systolic blood pressure (SBP) and diastolic blood pressure (DBP) were measured using a digital automatic blood pressure monitor (Dinamap model Pro100V2; Criticon GmbH, Norderstedt, Germany). Nonfasting venous blood samples were drawn and sent for biochemistry tests, including analysis of total cholesterol, high density lipoprotein cholesterol (HDL), low density lipoprotein (LDL) cholesterol, triglycerides, glucose, and HbA1c.

### English proficiency and other questionnaire-based measurements

A detailed interviewer-administered questionnaire was used to collect questions on demographics, acculturation, socioeconomic measures and reading literacy. The questionnaire was administered in three languages, including English, Tamil and Malay. English questionnaires were translated into the other two languages using a standard “forward-backward” translation procedure. Multilingual interviewers made the first contact with the participants and asked about participants’ language proficiency and preference for interview, and assigned those who preferred speaking Tamil or Malay and those who experienced difficulties in speaking English to the interviewers who were fluent in Tamil or Malay. The Malay-speaking sample was not included in this study, because of the relative smaller sample size (n = 226) and therefore limited statistical power.

Other collected information included age, sex, smoking history (0 = past or never; 1 = current), education (0 = secondary education or higher; 1 = primary education or lower), income (0 = earning > Singapore dollar [SGD] 1,000 per month; 1 = earning < SGD 1,000), current housing status (0 = 5 room flat/private house; 1 = 3-4 room flat or less), self-reported reading literacy (0 = adequate; 1 = inadequate) [20] and acculturation factors including length of residence in Singapore and country of birth (0 = Singapore-born; 1 = foreign-born).

### Statistical analysis

#### Logistic regression estimates

We developed univariate and multivariate logistic regression models to examine the associations between potential risk factors (e.g., age, sex, blood pressure, BMI, English proficiency, length of residence in Singapore, country of birth, literacy, and socioeconomic measures) and the presence of T2DM, DR, VTDR, or VI. Statistical analyses were performed using STATA software (Version 8.2, Stata Corp., College Station, TX). Interaction terms between English proficiency and socioeconomic measures were constructed and heterogeneity was tested with the Wald test; the significant term would be included in multivariate models.

#### Oaxaca decomposition

We used an Oaxaca decomposition method to decompose the differences in the prevalence of T2DM, DR, VTDR, and VI between the Tamil-speaking and English-speaking Indians. Oaxaca decomposition method is designed to decompose differences between in an outcome of interest into portions attributable to differences in the distributions of endowments (explanatory variables) and differences in returns to these endowments (coefficients) [21]. For example, this method has been widely in the labor market to examine whether the wage differences could be decomposed into characteristics (“explained”) and discriminations components (“unexplained”). Statistically, it allows us to decompose the difference between groups into two parts: Q and U. Q is the part of the outcome differential that is attributed to group differences in the covariates (e.g., the proportion of difference in prevalence of T2DM that can be explained by different levels of blood pressure in the two groups), and U is the part of the outcome differential that is attributed to discrimination or effects of differences in unobserved variables. The simple linear regression model can be expressed as:

(1) $$Y_{\ell }={X^{\prime }}_{\ell }\beta_{\ell }+\epsilon_{\ell },\;E\left(\epsilon_{\ell }\right)-0,\;\ell \in \left\{A,B\right\}$$

Where Y is the outcome variable; β is the coefficient and > is the error.

In the standard Blinder-Oaxaca decomposition, given group A (Tamil-speaking group) and group B (English-speaking group), the mean outcome difference R can be decomposed as:

(2) $$R=E\left(Y_{A}\right)-E\left(Y_{B}\right)=E{\left(X_{A}\right)}^{\prime }\beta_{A}-E{\left(X_{B}\right)}^{\prime }\beta_{B}$$

(3) $$\begin{array}{l} R=[E(X_{A})-E\left(X_{B}\right)]\beta *+[E{\left(X_{A}\right)}^{\prime }{\left(\beta_{A}-\beta *\right)}^{\prime }+E{\left(X_{B}\right)}^{\prime }(\beta *-\beta_{B})]\\ \;"\mathrm{explained}"\mathrm{part}(Q)\;"\mathrm{unexplained}"\mathrm{part}(U) \end{array}$$

Where β* is a flexible coefficient depends on the choice of reference group. We followed Neumark’s method where β*was derived from the pooled regression over both groups [22]. Since Y is a binary outcome (yes or no), we followed Fairlie’s method by setting (β) in Oaxaca logistic decomposition model [23].

## Results

**Figure 1:**
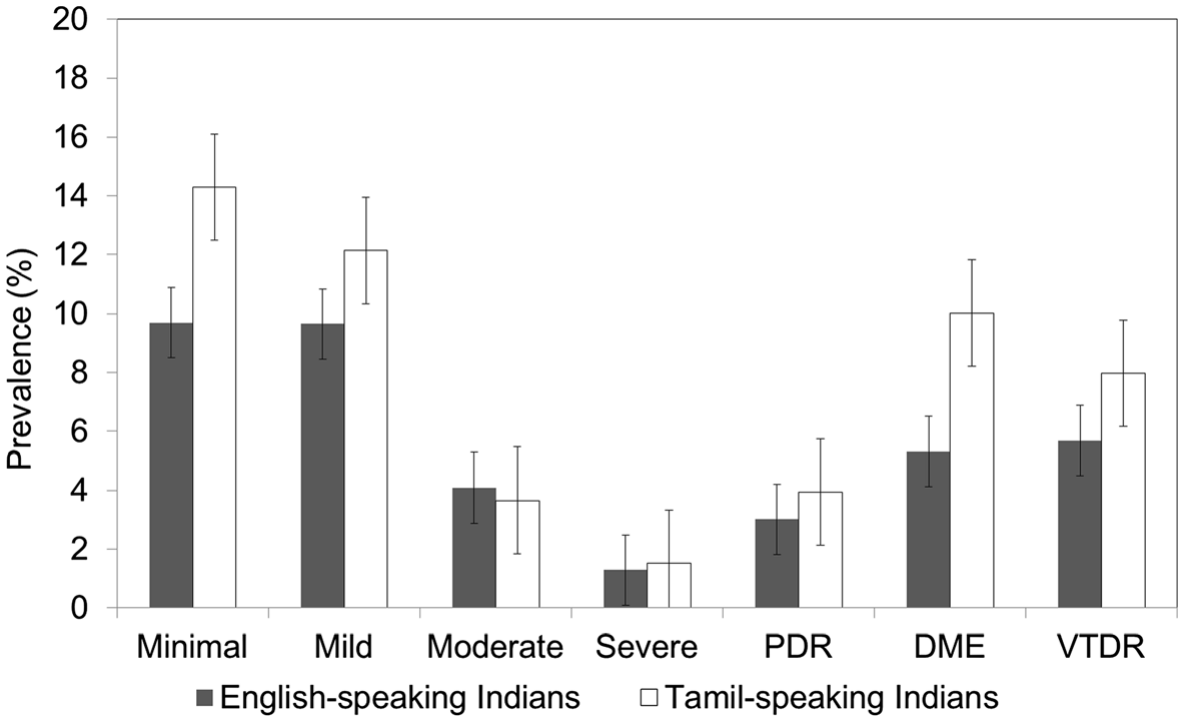
**Proportion of Diabetic Retinopathy Stratified by English Proficiency.** PDR = proliferative diabetic retinopathy; VTDR = vision-threatening diabetic retinopathy.

**Figure 2:**
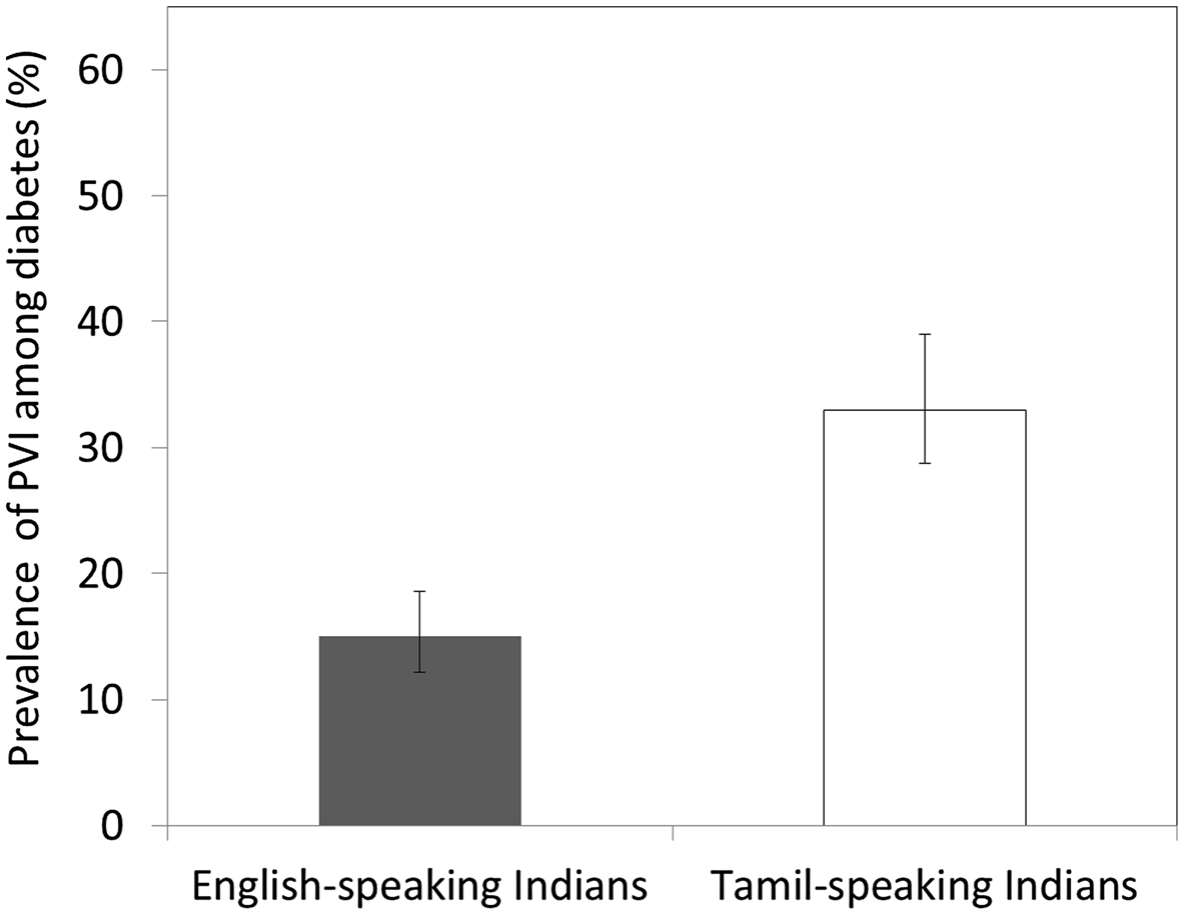
Proportion of Presenting Visual Impairment (PVI) Stratified by English Proficiency.

Table 1 shows the baseline characteristics of the 3,174 participants, stratified by their English proficiency. Compared with the English-speaking participants, Tamil-speaking participants were more likely to be older, female, non-smoker and born outside Singapore; and they had higher levels of BMI, HbA1c and SBP, and lower levels of DBP, socioeconomic status and literacy (all *P* < 0.05). Tamil-speaking Indians were more likely to have T2DM (raw prevalence: 46.2% versus 34.7%) and, among those with diabetes, DR (raw prevalence: 36.0% versus 30.6%), VTDR (raw prevalence: 11.0% versus 6.5%) and VI (raw prevalence: 32.4% versus 14.6%), compared with English-speaking Indians. Figure 1 shows the age-standardized prevalence of DR stratified by English proficiency and Figure 2 shows the age-standardized prevalence of VI.

**Table 1:** Sociodemographic and Clinical Characteristics of the Participants in the Singapore Indian Eye Study

		**English proficiency**		P value*
	**All participants (n = 3174)**	**English-speaking (n = 2289)**	**Tamil-speaking (n = 885)**	
Age groups				
40-49 years	866 (27.3)	750 (32.8)	116 (13.1)	
50-59 years	1036 (32.6)	818 (35.7)	218 (24.6)	
60-69 years	820 (25.8)	528 (23.1)	292 (33.0)	
70-80 years	452 (14.2)	193 (8.4)	259 (29.3)	<0.001
Gender (male)	1612 (50.8)	1300 (56.8)	312 (35.3)	<0.001
BMI (kg/m^2^)	26.1 (4.7)	26.0 (4.4)	26.4 (5.4)	<0.001
HbA1c (%)	6.4 (1.4)	6.4 (1.4)	6.6 (1.4)	0.002
SBP (mmHg)	134.9 (19.6)	132.9 (18.6)	140.2 (20.9)	<0.001
DBP (mmHg)	77.4 (10.1)	77.8 (10.1)	76.4 (10.1)	0.003
Total cholesterol (mmol/l)	5.2 (1.1)	5.2 (1.1)	5.1 (1.1)	<0.001
HDL cholesterol (mmol/l)	1.07 (0.32)	1.10 (0.32)	1.12 (0.31)	<0.001
LDL cholesterol (mmol/l)	3.33 (0.94)	3.36 (0.94)	3.23 (0.92)	0.003
Triglycerides (mmol/l)	1.96 (1.16)	2.01 (1.23)	1.83 (0.94)	<0.001
Current smoking (yes)	462 (14.6)	369 (16.1)	93 (10.5)	<0.001
Country of birth				
Foreign-born	1280 (40.3)	784 (34.3)	496 (56.0)	
Singapore-born	1894 (59.7)	1505 (65.8)	389 (44.0)	<0.001
Literacy level				
Adequate reading literacy	2941 (92.7)	2211 (96.6)	730 (82.5)	
Inadequate reading literacy	233 (7.3)	78 (3.4)	155 (17.5)	0.001
Education level				
Primary education or lower	1688 (53.3)	934 (40.9)	754 (85.2)	
Secondary education or higher	1482 (46.7)	1351 (59.1)	131 (14.8)	<0.001
Income level				
<S$1000	1538 (48.5)	881 (38.5)	657 (74.2)	
≥S$1000	1636 (51.5)	1408 (61.5)	228 (25.8)	<0.001
Housing type				
3-4 room flat or smaller	1996 (63.0)	1288 (56.3)	708 (80.0)	
5 room flat/private	1175 (37.0)	998 (43.7)	177 (20.0)	<0.001

Data presented are means (standard deviations) or number (%), as appropriate for variable. BMI = Body mass index; HbA1C = hemoglobin A1C; SBP = systolic blood pressure; DBP = diastolic blood pressure; HDL = high-density lipoprotein; LDL = low-density lipoprotein; S = Singapore dollar.

*P value, comparing the differences between English- and Tamil-speaking Indians, based on analysis of variance or chi-square test, as appropriate.

In traditional logistic regression model, after controlling for important covariates and risk factors, Tamil-speaking Indians were still significantly more likely to have T2DM (OR = 1.25; 95% CI: 1.04 to 1.52); and among those with diabetes, DR (OR = 1.20; 95% CI: 1.05 to 1.70), VTDR (OR = 1.70; 95% CI: 1.06 to 3.01) and VI (OR = 1.56; 95% CI: 1.03 to 2.34) compared to English-speaking Indians. There was no significant interaction between English proficiency and socioeconomic measures (*P* for interaction >0.05, data not shown) and between English-proficiency and age (*P* = 0.42). We also carried out stratified analyses by examining the associations of English proficiency (Tamil versus English) with T2DM, DR, VTDR, and VI, stratified by country of birth, education or income. The relationships between English proficiency and T2DM were slightly strengthened among the Singapore-born Indians, those with secondary education level or higher, and those with an income level < S$1000 (all with *P* < 0.001). The relationships of English proficiency with DR, VTDR and VI were slightly strengthened among the Singapore-born Indians, those with primary education level or less, and those with an income level ≥ S$1000 (all with *P* < 0.001).

Table 2 shows the findings of our Oaxaca decomposition analyses for T2DM, DR, VTDR and VI. Tamil-speaking Indians had a higher prevalence of T2DM than English-speaking Indians, by 11.6 percentage points. In the analyses stratified by age groups, Tamil-speaking Indians consistently had a higher prevalence of T2DM (data not shown), Two thirds of the difference (8.4/11.6) was attributed to the differences in the groups’ individual characteristics (“explained” component) and the rest could not be explained by the difference in individual characteristics (“unexplained” component). Age had the biggest contribution to the “explained” component: if age distributions in the two groups were similar, the difference in prevalence of T2DM would have been predicted to reduce by 3.4 percentage points. By contrast, if gender distributions in the two groups were similar, the difference in prevalence would have been predicted to even increase by 1.9 percentage points.

**Table 2:** Oaxaca Multivariate Decomposition of Language-related Disparities in the Presence of Type-2 Diabetes and Its Ocular Complications

	**Presence of Type-2 Diabetes**	**Presence of DR among those with diabetes**	**Presence of VTDR among those with diabetes**	**Presence of VI among those with diabetes**
	**Prediction (95%CI)**	**Prediction (95%CI)**	**Prediction (95%CI)**	**Prediction (95%CI)**
Prevalence in English-speaking Indians	**35.0% (33.0 to 36.9%)**	**30.1% (26.8 to 33.3%)**	**6.3% (4.6 to 8.0%)**	**16.7% (14.1 to 19.4%)**
Prevalence in Tamil-speaking Indians	**46.5% (43.2 to 49.9%)**	**36.1% (31.3 to 41.0%)**	**11.1% (8.1 to 14.2%)**	**33.7% (28.8 to 38.4%)**
Difference	**−11.6% (−15.5 to −7.6%)**	**−6.1% (−11.9 to −0.3%)**	**−4.9% (−8.4 to −1.3%)**	**−17.0% (−22.4 to −11.4%)**
Explained	**−8.4% (−11.3 to −5.7%)**	**−3.1% (−7.1 to −1.0%)**	**−1.2% (−3.3 to −0.9%)**	**−8.3% (−12.2 to −5.1%)**
Unexplained	**−3.2% (−7.7 to −1.4%)**	**−3.0% (−9.2 to −3.2%)**	**−3.7% (−7.5 to −0.2%)**	**−8.7% (−14.7 to −1.8%)**
Contribution of separate factors in explaining the explained proportion				
Demographic factors				
Age (year)	**−3.4% (−5.1 to −1.6%)**	**7.3% (2.2 to 12.3%)**	**2.6% (0.06 to 5.3%)**	**−4.0% (−6.9 to −0.9%)**
Gender (female vs. male)	**1.9% (0.9 to 2.9%)**	**3.7% (1.0 to 6.3%)**	0.9% (−0.3 to 2.1%)	1.2% (−1.2 to 3.0%)
Systemic biological factors				
BMI (kg/m^2^)	−0.6% (−1.2 to 0.03%)	0.01% (−0.3 to 0.4%)	0.01% (−0.1 to 0.1%)	0.01% (−0.2 to 0.2%)
SBP (mmHg)	**−3.5% (−4.7 to −2.2%)**	**−3.1% (−5.1 to −1.1%)**	−0.6% (−1.4 to 0.1%)	−0.2% (−0.7 to 1.3%)
DBP (mmHg)	**−0.8% (−1.4 to −0.2%)**	**−1.5% (−2.8 to −0.2%)**	−0.5% (−1.1 to 0.1%)	−0.5% (−1.1 to 0.4%)
HDL (mmol/l)	0.3% (−0.02 to 0.6%)	−0.6% (−1.4 to 0.2%)	−0.1% (−0.5 to 0.2%)	−0.6% (−1.2 to 0.1%)
LDL (mmol/l)	**−1.4% (−2.2 to −0.6%)**	−0.4% (−1.0 to 0.2%)	0.1% (−0.2 to 0.2%)	0.3% (−0.2 to 0.8%)
Triglyceride (mmol/l)	**0.7% (0.2 to 1.1%)**	−0.1% (−0.9 to 0.7%)	0.1% (−0.4 to 0.5%)	0.2% (−0.4 to 0.9%)
Hba1c (%)	-	0.9% (−0.3 to 2.2%)	0.2% (−0.01 to 0.6%)	0.2% (−0.1 to 0.7%)
Duration of diabetes (year)	-	**−5.1% (−7.5 to −2.7%)**	**−1.7% (−3.1 to −0.3%)**	−0.5% (−1.2 to 0.2%)
Health related behaviors				
Smoking	−0.3% (−0.6 to 0.04%)	−0.2% (−0.5 to 0.2%)	−0.1% (−0.4 to 0.1)	0.01% (−0.2 to 0.3%)
Alcohol	−0.01% (−1.2 to 1.0%)	0.2% (−0.6 to 1.1%)	−0.1% (−0.4 to 0.2)	−0.3% (−0.9 to 0.5%)
Acculturation factors				
Country of birth	**2.1% (0.4 to 3.7%)**	−0.5% (−2.5 to 1.4%)	0.1% (−0.9 to 0.9%)	−0.1% (−2.6 to 1.1%)
Duration of residency (year)	−1.9% (−4.0 to 0.01%)	−0.3% (−1.3 to 0.8%)	0.1% (−0.4 to 0.6%)	−0.2% (−1.1 to 0.7%)
Socioeconomic factors				
Reading literacy	0.4% (−0.4 to 1.4%)	−0.9% (−2.5 to 0.7%)	−0.2% (−0.7 to 0.6%)	**−2.0% (−3.3 to −0.7%)**
Education	−0.9% (−2.5 to 1.0%)	1.3% (−1.7 to 4.3%)	0.3% (−1.1 to 1.9%)	−1.1% (−4.0 to 1.3%)
Income	−0.4% (−2.0 to 0.9%)	**−2.2% (−4.7 to −0.2%)**	**−1.4% (−3.0 to −0.2%)**	**−0.6% (−5.8 to −0.1%)**
Housing type	−0.5% (−1.3 to 0.4%)	**−1.7% (−3.2 to −0.01%)**	**−1.0% (−1.8 to −0.2%)**	−0.3% (−2.2 to 0.7%)

95% CI = 95% confidence interval; DR = diabetic retinopathy; VTDR = vision-threatening diabetic retinopathy; VI = visual impairment; BMI = body mass index; SBP = systolic blood pressure; DBP = diastolic blood pressure; HDL = high-density lipoprotein; LDL = low-density lipoprotein; Hba1c = hemoglobin A1C. Bold font highlights statistical significance (P < 0.05). Smoking category: 0 = current; 1 = never; Alcohol category: 0 = current; 1 = never; Country of birth category: 0 = foreign-born, 1 = Singapore-born; Reading literacy category” 0 = adequate, 1 = inadequate; Education category: 0 = secondary education or higher, 1 = formal education or lower; Income category: 0 = lower than SGD$1,000, 1 = SGD$1,000 or more; Housing type category: 0 = 5-room flat or bigger, 1 = 4-room flat or smaller.

Among the patients with T2DM, Tamil-speaking Indians were more likely to have DR (by 6.1 percentage points) and VTDR (by 4.9 percentage points) than English-speaking Indians (Table 2). 50.8% (3.1/6.1) of the difference in DR prevalence and 24.5% (1.2/4.9) of the difference in VTDR were attributed to the differences in the groups’ individual characteristics (“explained” component) and the rest could not be explained by the difference in individual characteristics (“unexplained” component). Duration of diabetes and socioeconomic status (including income and housing type) had substantial contribution to the “explained” component for both DR and VTDR prevalence.

Among the patients with T2DM, Tamil-speaking Indians were twice as likely as English-speaking Indians to have VI, giving a gap of 17.0 percentage points (Table 2). Around 50% (8.3/17.0) this difference was attributed to the differences in the groups’ individual characteristics (“explained” component). Age and socioeconomic factors (including reading literacy and income) had substantial contribution to the “explained” component.

To avoid over-adjustment, we also carried out supplementary analyses in Oaxaca decomposition model by controlling only those independent variables that were statistically significant in univariate regression analyses. First, we found that 53.9% (6.2/11.6) of the language-related disparity in prevalence of T2DM was attributed to “explained” component, and 46.1% (5.4/11.6) to “unexplained” component, after controlling for the effect of age, gender, SBP, DBP, LDL, triglyceride, and country of birth. Second, 53.8% (2.7/5.1) of the language-related disparity in prevalence of DR (among those with T2DM) was attributed to “explained” component, and 46.2% (2.3/5.1) to “unexplained” component, after controlling for the effect of age, gender, SBP, DBP, duration of diabetes, income and housing type. Third, 38.9% (1.8/4.6) of the language-related disparity in prevalence of VTDR (among those with T2DM) was attributed to “explained” component, and 61.1% (2.8/4.6) to “unexplained” component, after controlling for the effect of age, duration of diabetes, income, and housing type. Finally, 46.9% (9.0/19.2) of the language-related disparity in prevalence of VI (among those with T2DM) was attributed to “explained” component, and 53.1% (10.2/19.2) to “unexplained” component, after controlling for the effect of age, reading literacy and income. None of the independent variables has significant influence on “unexplained” component (data not shown).

## Discussion

This is the first population-based assessment of the association of English proficiency with T2DM and its key ocular complications. We demonstrated that there were significant language-related disparities between persons who were Tamil-speaking and English speaking: Tamil-speaking Indians were more likely to have T2DM than English-speaking Indians and, among those with diabetes, more likely to DR, VTDR and VI, complications which have immediate and substantial impacts on a patient’s quality of life. Oaxaca decomposition method is an established tool for macroeconomic analysis and it provided us with a unique opportunity to identify factors explaining language-related disparities in Asian Indians living in a culturally diverse modern society [21]. For the prevalence of T2DM, it was age and systemic biological factors such as blood pressure and LDL that accounted for a substantial proportion of language-related disparity (Table 2). Surprisingly, socioeconomic and acculturation factors had limited contribution, suggesting that the influence of language on T2DM prevalence was not mediated by different levels of socioeconomic status. The implication is that reducing socioeconomic differences alone may be unlikely to remove language-related disparities in the prevalence of T2DM. These findings are critically important in developing policies and implementing linguistic-specific programs in the prevention of diabetes in Asia’s multi-linguistic societies. Among those with diabetes, however, socioeconomic measure had significant contribution to the language-related disparities in prevalence of DR and VTDR. These findings reflect the complex influences of socioeconomic measures on the prevention and management of diabetes ocular complications. 

The origins of the “unexplained” language-related disparities are multi-factorial, and as suggested by Marmot and others, the disparities could be broadly due to material deprivation and/or the lack of capability to control life and fully participate in the society (psychosocial disadvantage) [24]. We propose two possible explanations. First, English proficiency can be perceived as a proxy measure of acculturation and reflects immigrants’ culture, social identity and political ideology [9], given that most of our participants are first or second generation of the immigrants from Indian subcontinent. In this regard, Asian Indians who speak English during interview are presumably the ones who are more adaptive to local culture and are more likely to be absorbed into the dominant society – a community that have an advantage in obtaining occupation opportunity, receiving social support, avoiding psychological stressors, and maintaining a healthy lifestyle. As a result, they may be less likely to have diabetes and its complications compared to Tamil-speaking Indians. Second, the “unexplained” disparities may be due to a lack of diabetes knowledge, medical information, patient-physician communication, and treatment adherence among those with poor language skill [6-8,25]. This view is supported by the findings from the United States that language ability can directly influence access to health care and has impact on health among the Hispanic populations [6-8]. Finally, our findings may be attributable to a “healthy migrant effect” (i.e., the new immigrants were generally healthier than the local residents), but our stratified analyses showed that this language-related disparity was also seen in Singapore born Indians. Further research is needed to evaluate and identify ways in which language barriers affect diabetes management and DR care, and to assess the cost effectiveness of language-specific health improvement programs and linguistic service among this heterogeneous population. Geographic condition is unlikely an explanation, given that the two communities were living in the same areas (totaling 42.6 sq mile) and there were no transformational barriers across different districts [16].

The strengths of this study include its population-based nature, objective measurement of diabetes and DR, the use of Oaxaca decomposition analysis, and the ability to adjust for a wide range of potential risk factors. Several limitations should be highlighted as well. First, while interview language has been shown to be a better acculturation indicator than self-reported English proficiency [10], we could not exclude the possibility that there were some Indians who were proficient in English but chose/preferred to respond in Tamil, and consequently the observed associations may be biased towards the null. Nevertheless, we have opted to use the term “English proficiency” rather than “language preference”; although one is invariably linked with the other, the choice of Tamil language is more of an indicator of a lack of English language proficiency in this society. Second, our findings may be cultural specific, and not be generalizable to other Asian populations and other languages. Third, we did not collect data regarding diet, physical activity, and detailed use of medication, and the lack of these information may have led to an overestimation of language-related disparities. Finally, the effect of acculturation has been considered in our multivariate analysis by including migration status and length of residence in Singapore as covariates, but we did not consider the effects of other potential cultural factors (e.g., cultural traditions and behaviors).

## Conclusions

In summary, in a society where English is the predominant working language, Tamil-speaking Indians are more likely to have T2DM and eye complications (DR and VI) than English-speaking Indians. The language-related disparities cannot be fully explained by biological risk factors and traditional socioeconomic measures. Language represents one of the key social determinants of health in many new multilingual societies around the world, including United States, Europe and Asia. While the pathways through which English language proficiency affect health remains to be determined, the immediate application of our study suggests that language service itself should be recognized as a critical component of health equality and health care programs.

## Competing interests

The authors declare that they have no competing interests.

## Authors’ contributions

Y.Z. conducted the statistical analysis and wrote the manuscript; E.L.L., directed the implementation of the study and helped reviewed the manuscript; P.C. helped conduct statistical analysis and edited the manuscript; A.R.A. helped designed the analytic strategy; J.D. contributed to discussion and helped review the manuscript; J.J.W. helped supervise the field activities and edited the manuscript; P.M. helped conduct the literature review and edited the manuscript; E.T. helped set up and supervise the project and reviewed the manuscript; T.Y.W. designed and supervised the project, contributed to discussion, and reviewed/edited the manuscript. All authors read and approved the final manuscript.

## Pre-publication history

The pre-publication history for this paper can be accessed here:

http://www.biomedcentral.com/1471-2458/12/781/prepub
